# Dietary Diversity, Micronutrient Adequacy and Bone Status during Pregnancy: A Study in Urban China from 2019 to 2020

**DOI:** 10.3390/nu14214690

**Published:** 2022-11-05

**Authors:** Wuxian Zhong, Ai Zhao, Hanglian Lan, Shuai Mao, Pin Li, Hua Jiang, Peiyu Wang, Ignatius Man-Yau Szeto, Yumei Zhang

**Affiliations:** 1 Department of Nutrition and Food Hygiene, School of Public Health, Peking University Health Science Center, Beijing 100191, China; 2 Vanke School of Public Health, Tsinghua University, Beijing 100084, China; 3 Yili Maternal and Infant Nutrition Institute, Beijing 100071, China; 4 Inner Mongolia Dairy Technology Research Institute Co., Ltd., Hohhot 010110, China; 5 School of Nursing, Peking University Health Science Center, Beijing 100191, China; 6 Beijing Key Laboratory of Toxicological Research and Risk Assessment for Food Safety, School of Public Health, Peking University Health Science Center, Beijing 100191, China; 7 Department of Social Medicine and Health Education, School of Public Health, Peking University Health Science Center, Beijing 100191, China

**Keywords:** dietary quality, minimum dietary diversity for women, nutrient adequacy, bone health, speed of sound, pregnancy

## Abstract

Diet quality during reproduction is crucial to maternal and infant health. However, the association between dietary diversity and bone health of pregnant women remains unclear. We aimed to evaluate the dietary quality of Chinese urban gravidas using the dietary diversity score (DDS), and to explore the relationship of the DDS with micronutrient adequacy and bone health. In this cross-sectional study, we analyzed data from 775 pregnant women aged 18 years or older in urban China. Dietary diversity was assessed using the Minimum Dietary Diversity for Women (MDD-W) indicator. A 24-h dietary recall was used to collect diet data and to calculate the MDD-W and the nutrient intake. Bone health was measured using quantitative ultrasound and assessed by the speed of sound (SOS). Pearson’s correlation coefficients between the DDS and the nutrient adequacy ratio (NAR) were calculated. A multivariable linear regression model was used to estimate the effect size of the DDS on the SOS. The mean DDS was 6.61 ± 1.53 points and 91.0% of participants reported the consumption of five or more food groups. Women in the diverse diet subgroup (DDS ≥ 7 points) were more likely to consume all kinds of food except starchy staples and had higher NARs. Pearson’s correlation coefficients between the DDS and the NAR ranged from 0.161 to 0.484. For participants in the second trimester, those with a diverse diet had a higher SOS. A multivariable linear regression analysis showed that the DDS was positively and significantly associated with the SOS (β = 17.18, 95% CI = 5.97–28.39, *p* = 0.003), but this was not the case for women in the first and third trimesters. Urban Chinese women had good dietary diversity during pregnancy. A higher dietary diversity was associated with a higher NAR. From the point of view of bone, a diverse diet was positively correlated with better bone status, suggesting the importance of improving diet diversity for pregnant women, especially from mid-pregnancy.

## 1. Introduction

Pregnancy is an important and vulnerable period for women and their infants, with nutrition being the cornerstone of both maternal and infant health. During this stage, a varied and balanced diet containing comprehensive nutrients is recommended to protect against maternal malnutrition and to support offspring development [1]. Previous studies have revealed that deficiency in micronutrients such as vitamin A, vitamin D and iron among pregnant women is still a public health issue in China [2,3,4]. The *China Nutrition and Health Surveillance (CNHS)* 2015–2017 revealed that the prevalence of vitamin A deficiency was 1.2% and 10.5% for marginal deficiency in rural pregnant women [2], respectively. In 2015–2017, 41.96% of pregnant women had vitamin D deficiency, and 45.46% had vitamin D insufficiency [3], although vitamin D levels could be mediated by the amount of sunlight. Another large multicenter study found that the overall prevalence of anemia during pregnancy was 23.5% [4]. Furthermore, poor prenatal dietary quality is associated with higher risk of preterm birth, low birthweight, underweight, total congenital heart defects and ventricular septal defects [5,6,7].

The dietary diversity score (DDS) is a useful and convenient dietary assessment tool used in large-scale surveys and in varied populations [8,9]. It reflects dietary quality by quantifying the number of food items or groups based on dietary guidelines or by self-report, with a higher score indicating a more varied diet. The Minimum Dietary Diversity for Women of reproductive age (MDD-W) is a food group diversity indicator proposed by the Food and Agriculture Organization (FAO) [10]. It has been correlated with micronutrient adequacy [11,12], but the dietary diversity assessed by the MDD-W and its correlation with nutrient adequacy in Chinese pregnant women remains unclear.

In recent years, several studies have explored the association between dietary diversity and metabolic diseases such as hypertension, diabetes and obesity [13,14,15,16]. However, little attention has been paid to the relationship between dietary diversity and bone health in pregnant women. Women in conception have increased demands for nutrients, but mothers might not improve their diet after pregnancy [17]. Although physiological adaptions such as increased intestinal calcium absorption exist [18] to supply the required calcium for fetal skeleton development, insufficient dietary intake can result in the transfer of calcium from bone tissue into blood. Previous studies have suggested that the stiffness index (an indicator of fracture risk) and bone mass decline significantly during pregnancy and lactation [19,20], which in severe cases can cause pregnancy- and lactation-associated osteoporosis [21]. Other controlled trials have found that calcium supplementation helps to reduce bone resorption during pregnancy and improve postpartum bone recovery [22,23]. Given that women of reproductive age are also in the stage of accumulating peak bone mass, diet intervention during pregnancy may exert a protective and beneficial effect on long-term skeleton health.

Therefore, this current cross-sectional study aimed to: (1) evaluate the dietary quality of Chinese pregnant women using the MDD-W and assess the correlation of the MDD-W with micronutrient adequacy; and (2) explore the association between the MDD-W and bone health measured by quantitative ultrasound (QUS).

## 2. Methods

### 2.1. Study Population

The present study makes use of data from the Young Investigation (YI) study [24], a cross-sectional survey conducted from 2019 to 2020 that aimed to investigate the health and nutrition status of pregnant women, lactating women and young children aged 0–3 years. In the YI study, ten cities were selected according to geographical location and economic level: Beijing, Guangzhou, Suzhou, Chengdu, Shenyang, Ningbo, Nanchang, Lanzhou, Hohhot and Xuchang. Within each city, one hospital or hospital-based maternal and child healthcare center was chosen as an investigation center and at least 30 eligible gravidas of each trimester were surveyed by convenience sampling. As a part of the YI study, which aimed to investigate the nutrition status of pregnant women, a total of 934 women completed the survey. The YI study was approved by institutional review boards at the Peking University (NO. IRB00001052-19045). Written informed consent was obtained from all participants before the interviews.

In current study, the inclusion criteria were: healthy 18–45-year-old women who had a singleton pregnancy. The exclusion criteria were: women with dysmnesia or psychiatric disorders [25], or those with infectious diseases (pulmonary tuberculosis, viral hepatitis or human immunodeficiency virus infection) or without data on bone status. Finally, 775 pregnant women were included (as shown in Supplementary Material Figure S1). The proportion of consuming a diverse diet was estimated as 85% based on a study conducted in Northwest China [7], with an allowable error of 5% and standard normal deviation at a confidence limit of 95%. The final calculated sample size was 216 with the addition of a 10% non-response rate for each trimester.

### 2.2. Dietary Assessment

Data were collected by a face-to-face questionnaire survey. The unified training of interviewers was completed before the start of study. The one-time 24-h dietary recall, a dietary assessment method commonly used in nutritional epidemiology [5,12,26,27,28,29,30], was used to assess all foods and beverages consumed over the previous day, and standard-sized bowls, teaspoons and illustrated photos of food items were shown in order to improve the estimation accuracy. Total energy and micronutrient intake were calculated based on the Chinese Food Composition Table [31]. The dietary recall provided intake values for vitamin A, thiamin, riboflavin, niacin, vitamin C, vitamin E, folate, calcium, phosphorus, potassium, magnesium, iron, zinc, copper, and selenium.

Based on the diet data collected, the DDS was computed according to guidance provided by the FAO for the MDD-W [10] at an individual level. As proposed by the FAO, the MDD-W contains ten food groups: starchy staples (grains, white roots and tubers, and plantains); pulses (beans, peas and lentils); nuts and seeds; dairy; flesh foods (meat, poultry and fish); eggs; vitamin A-rich dark green leafy vegetables; other vitamin A-rich fruits and vegetables; other vegetables; and other fruits [10]. Consumption over the past 24 h was assigned 1 point for each food group and no minimum weight restriction was considered. For each individual, a minimum of 0 and a maximum of 10 points could be obtained, with higher scores indicating higher dietary diversity.

To estimate the nutrient adequacy of the diet, the nutrient adequacy ratio (NAR) [32] was calculated by dividing the participants’ actual intakes of each micronutrient from the food by the estimated average requirement (EAR) of that nutrient for the corresponding pregnancy stage [33]. In these analyses, the micronutrient values from supplements were not added to the reported dietary intake calculation because the MDD-W was an indicator for foods. The mean adequacy ratio (MAR) was calculated as the mean of all NARs. The NARs were truncated at 1 to avoid situations where nutrients with a high NAR compensate for nutrients with a low NAR. Therefore, both the NAR and the MAR range from 0 to 1.

### 2.3. Anthropometric Measurements

Considering the radioactivity of dual-energy X-ray absorptiometry (DXA), bone status was measured at the distal third radius of the non-dominant hand using QUS (Sunlight MiniOmni Bone Sonometer, BeamMed Ltd., Petah-Tikva, Israel), and the speed of sound (SOS, m/s) was obtained. Previous studies have shown that SOS measurement by QUS correlated well with bone mineral density (BMD) measurement by DXA [34,35]. The T-score and the Z-score can be computed based on the SOS. However, both of them were obtained using a Hispanic population as a reference and consequently were not appropriate for the evaluation of Chinese pregnant women. Accordingly, in the present study we chose the SOS as a dependent variable, because higher SOS indicates better bone quality. Body Weight and height were measured at the time of interview, weight was measured using a digital weight scale without shoes and wearing minimal clothes, to the nearest 0.01 kg, and height was measured to the nearest 0.1 cm using a stadiometer with a fixed vertical backboard and an adjustable head piece (Seca, Hamburg, Germany). The body mass index (BMI, kg/m^2^) was calculated and pre-pregnancy BMI was categorized as underweight or normal (BMI < 24 kg/m^2^), overweight (24 ≤ BMI < 28 kg/m^2^), and obese (BMI ≥ 28 kg/m^2^) [36].

### 2.4. Covariates

A self-designed questionnaire on sociodemographic characteristics, reproductive history and lifestyle data was used and completed by trained investigators. The following covariates were chosen according to previous literature [37,38,39]: age (18–24, 25–34 and ≥35 years), nationality (Han or minority), area (South or North), parity (nulliparous or multiparous), education level (junior middle school or below; senior high school or secondary specialized school; undergraduate college or above), monthly family income per capita (<5000, 5000–9999 and ≥10,000 yuan, according to the national average level from National Bureau of Statistics of China), daily energy intake, smoking status (never, stopped or still smoking), second-hand smoke exposure (“yes” or “no”, defined as exposure to tobacco smoke from someone nearby for ≥15 min/day), alcohol use (never, stopped or still drinking), BMI, and physical activity level. The validated Chinese version of the International Physical Activity Questionnaire—Short Form (IPAQ-SF) [40,41] was used to assess physical activity (PA). The participants reported frequencies and duration of PA during the past week. PA was divided into three intensities: vigorous, moderate and walking. Intensity was measured using the metabolic equivalents (METs), and MET-minutes per week (MET-mins/week) were calculated and divided into low, medium and high level [42]. Calcium supplement intake (indicated by “yes” or “no”) was collected using a self-designed nutrient supplementation table.

### 2.5. Statistical Analysis

We first described and examined the participants’ sociodemographic characteristics in different trimesters. One-way analysis of variance (ANOVA) and the Kruskal-Wallis test were used to compare values between groups for continuous variables, whereas chi-square and Fisher’s exact tests were used for categorical variables. Considering the distribution of the DDS in the current sample, a cut-off value of 7 (median of the DDS) rather than 5 [10] was used to classify participants into diverse or non-diverse groups. We examined the correlation between the DDS and the NAR using Pearson’s correlation coefficient. A multivariable linear regression model was used to estimate the effect size of the DDS (as a continuous variable) on the SOS; the coefficient was reported in a crude and adjusted model, controlling for the factors mentioned above. All statistical analyses were performed using SAS software version 9.4 (SAS Inc., Cary, NC, USA), and statistical significance was defined as two-sided *p* < 0.05.

## 3. Results

The descriptive characteristics of the subjects are detailed in Table 1. In brief, the population of the current study was mainly middle-aged pregnant women with relatively high education and economic levels and good dietary diversity (the median DDS was 7); 91.0% of participants reported the consumption of five or more food groups. Women in the third trimester have the highest BMI and a higher proportion of medium physical activity and calcium supplement intake, whereas those in the first trimester have a higher pre-pregnancy BMI but the lowest DDS.

**Figure 1:**
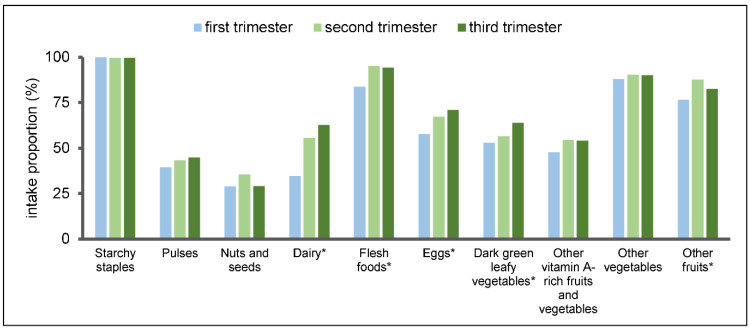
Proportions of food group consumption in the different trimesters. * Significant difference of food group intake proportion in the three trimesters (*p* < 0.05).

Figure 1 shows the intake proportions for participants in the different trimesters according to the food groups of the MDD-W. Women in early pregnancy are less likely to consume dairy (34.5%), flesh foods (83.7%), eggs (57.6%), dark green leafy vegetables (53.0%) and other fruits (76.5%) compared to women in the other two trimesters (detailed proportions of food group consumption in the different trimesters can be found in Supplementary Material Table S1). In general, the top five food groups with the highest consumption percentage are starchy staples, flesh foods, other vegetables, other fruits and eggs, as seen in Table 2. In addition, there were significantly more subjects in the diverse diet subgroup consuming all kinds of food except starchy staples. Among the women with a non-diverse diet, less than 50% have eaten eggs, dark green leafy vegetables, other vitamin A-rich fruits and vegetables, dairy, pulses and nuts/seeds over the past 24 h.

Of the 15 nutrients assessed, only folate showed a mean intake below 60% of the EAR in women with a diverse diet, whereas folate, calcium, vitamin A, riboflavin and vitamin C all showed a mean NAR of <0.60 in women with a non-diverse diet. Table 3 compares the NARs for participants with non-diverse and diverse diets. For all the nutrients, the NARs were significantly higher in those consuming more food groups. Pearson’s correlation analysis showed that all the NARs correlated positively and significantly with the DDS, ranging from 0.161 to 0.484 (Pearson’s correlation coefficients between the NAR and the DDS in the different trimesters were shown in Supplementary Material Table S2).

We then analyzed the relationship between diverse diet and bone status in different populations (Table 4). Women in the second trimester and with a diverse diet showed higher SOS, T-scores and Z-scores compared to those with a non-diverse diet. For gravidas in their early or late trimesters, having a diverse diet was not associated with bone status.

Finally, a multivariable linear regression model was used to estimate the effect size of the DDS on the SOS. Univariate regression analysis showed that the DDS was positively associated with the SOS of women in their second trimester (β = 16.55, *p* = 0.002) and of all participants (β = 8.39, *p* = 0.008). After adjusting for age, nationality, area, education level, income level, parity, current BMI, physical activity level, energy intake, smoking status, second-hand smoke exposure and alcohol use, the correlation was attenuated for all participants (β = 8.04, *p* = 0.022) but strengthened for women in their second trimester (β = 18.07, *p* = 0.002). Further adjustment for calcium supplement intake obtained similar results, as seen in Table 5.

## 4. Discussion

In this study, we found that Chinese urban pregnant women had a relatively high dietary diversity. The mean NAR was moderate and significantly higher in participants with a diverse diet. For women in the second trimester, the DDS was positively associated with the SOS, indicating the importance of improving dietary diversity to promote maternal bone health.

Judged by the standard cut-off value of the MDD-W, 55.0% of gravidas in Nepal and 57.7% of non-lactating and non-pregnant women of reproductive age in Latin America reported a diverse diet [26,43], and 70.3% of pregnant women in Northwest China reached the MDD-W cut-off [7], which is lower than the 91.0% of the current study. This suggested dietary diversity during reproduction may not be a major issue for Chinese urban women. However, we also noticed that the DDS in the first trimester was significantly lower than that in later pregnancy, with less dairy, eggs, flesh foods, dark green leafy vegetables and other fruits likely to be consumed. This was consistent with previous research reporting that nausea and vomiting affected food intake and diet quality from before to early pregnancy but not in late pregnancy [44]. This result could be explained by the common occurrence of morning sickness during the first trimester, which affects appetite, suggesting the urgency of early intervention to improve dietary diversity.

Similar to previous studies [7,26], we found that gravidas with an inadequate DDS had lower intake proportions of all food groups except starchy staples. Notably, less than 50% of pregnant women in both groups consumed nuts and seeds in the past 24 h, which are a good source of protein and unsaturated fatty acids. In addition, less than half of women in the non-diverse diet group ate foods that are typically recommended as part of a healthy dietary pattern, such as pulses and dairy [45]. In terms of micronutrient intake, we found that women with a diverse diet had statistically significantly higher NARs compared to those with a non-diverse diet, and the DDS was positively and moderately associated with the MAR, which was consistent with previous research [7,26,43], providing evidence for its applicability in pregnant women. Furthermore, it is worth noting that in the present sample some nutrients showed a mean NAR lower than 60%. This may be due to the fact that micronutrients from supplements were not taken into account when calculating the NAR, and nutrient supplement usage among pregnancy was quite prevalent in China [46]. Given that foods are nutritionally diverse, have high biological value and are low risk [47,48], education and the promotion of a diverse diet has become an important way to improve maternal micronutrient status [49].

Previous studies have explored the relationship between dietary diversity and bone health in other populations. A cross-sectional study [50] found that the DDS (based on five food groups) was positively associated with femoral neck BMD in women aged 20–25 years, but the association was attenuated after adjustment for mean energy intake. Another cohort study [8] revealed that a higher DDS contributed to a lower risk of fracture in women aged 40–60 years. Consistent with these studies, our study also found that higher dietary diversity was correlated with better SOS among women in the second trimester. There are several potential pathways listed below that may link diet to bone health. On the one hand, the DDS was positively associated with the MAR and the NAR, showing that a diverse diet provides more comprehensive and sufficient nutrition. Women with a higher DDS were found to have a higher intake of animal foods, including dairy, eggs and flesh foods. These are good sources of nutrients such as calcium, vitamin D and protein, which have been shown to be beneficial for peak bone mass, decreased bone resorption and increased BMD [22,51,52,53]. On the other hand, we found that women with a diverse diet had a higher consumption of vegetables, fruits, pulses and nuts/seeds, which are rich in unsaturated fatty and bioactive compounds such as flavonoids, protecting against oxidative stress and inflammation [54,55,56,57].

Furthermore, the stratified analysis showed that dietary diversity in first- and third-trimester women was not associated with the SOS. Women at different pregnancy stages may have varied lifestyles and physiological status, resulting in this heterogeneity. As mentioned above, calcium is important for the development of peak bone mass and suppressed bone resorption [22,51]. A prospective cohort study [19] reported that bone loss occurred from the second to the third trimester, suggesting that from the second trimester onwards, calcium demand increased greatly but a huge intake gap existed, leading to a significant loss of bone calcium and complaints such as leg cramps [58]. Another possible reason might be that during the third trimester, women had higher proportions and amounts of calcium supplement. However, further adjustment for calcium supplement intake (indicated by “yes” or “no”) in the regression analysis obtained similar results. It should be noted that the duration and amount of calcium supplementation could also have a large impact. In the future, more research is needed to elucidate this uniqueness and the underlying mechanism. Moreover, our findings also indicated that the second trimester might be a window period for dietary intervention to accumulate sufficient calcium reserve.

### Strengths and Limitations

To our best knowledge, this is the first study to focus on the association between dietary diversity and bone quality in pregnant women. However, some limitations also exist. Firstly, our study is cross-sectional and therefore unmeasured confounding cannot be completely ruled out, despite controlling the covariates as much as possible. Secondly, similar to previous studies [5,59], a one-time 24-h dietary recall was used for simplicity, which might cause the intraindividual variation of nutrient intake and confound the correlations between the DDS and NAR, and interpretation of the results should be done with caution. In future studies, different dietary assessment tools or repeated food recalls in at least 20% of the sample should be adopted. Moreover, a recent study [32] that calculated both the 1- and 3-day DDS obtained similar results. Therefore, we believe that the effect of DDS on bone health is still credible. Thirdly, the MDD-W may not give enough weight to the food groups that benefit bone health and consequently may attenuate the relationship between diet and bone quality. Fourthly, due to the limited QUS reference database for Chinese pregnant women, we cannot explore the appropriate cut-off value of the MDD-W for better bone health in the current population. In addition, considering that the radiation from DXA is not suitable for pregnant women, in the current study we used QUS, a reliable and more convenient method [60], rather than the gold standard method. Lastly, because of the specialized population, we could not collect adequate blood samples to detect bone metabolism indicators, which limited the analysis dimension of the DDS in relation to bone health.

## 5. Conclusions

Urban Chinese women had good dietary diversity during pregnancy. A varied diet was recommended to achieve a higher NAR and MAR. From the point of view of bone health, diverse diets were positively associated with better bone status independent of calcium supplement intake, suggesting the importance of encouraging diet diversity for gravidas, especially from mid-pregnancy.

## Figures and Tables

**Table 1:** Sociodemographic characteristics of women in the different trimesters ^a^.

	First Trimester (*n* = 264)	Second Trimester (*n* = 259)	Third Trimester (*n* = 252)	All (*n* = 775)	*p*
Age (years)	29.2 ± 4.5	29.0 ± 4.0	29.9 ± 4.5	29.4 ± 4.3	0.061
Age group (years)					
18–24	39 (14.8)	39 (15.1)	28 (11.1)	106 (13.7)	0.242
25–34	190 (72.0)	198 (76.5)	191 (75.8)	579 (74.7)	
35–44	35 (13.3)	22 (8.5)	33 (13.1)	90 (11.6)	
Han nationality	242 (91.7)	244 (94.2)	235 (93.3)	721 (93.0)	0.514
South area	152 (57.6)	160 (61.8)	147 (58.3)	459 (59.2)	0.583
Currently working	166 (62.9)	158 (61.0)	145 (57.5)	469 (60.5)	0.482
Education level					
Basic	37 (14.0)	40 (15.4)	26 (10.3)	103 (13.3)	0.179
Secondary	52 (19.7)	36 (13.9)	40 (15.9)	128 (16.5)	
Higher	175 (66.3)	182 (70.3)	184 (73.0)	541 (69.8)	
Monthly income (yuan)					
0–4999	84 (31.8)	78 (30.1)	68 (27.0)	230 (29.7)	0.746
5000–9999	124 (47.0)	125 (48.3)	124 (49.2)	373 (48.1)	
≥10,000	48 (18.2)	55 (21.2)	54 (21.4)	157 (20.3)	
Multiparous	107 (40.5)	93 (35.9)	100 (39.7)	300 (38.7)	0.592
Smoking status					
Never smoked	247 (93.6)	245 (94.6)	242 (96.0)	734 (94.7)	0.714
Stopped smoking	16 (6.1)	12 (4.6)	9 (3.6)	37 (4.8)	
Smoking during pregnancy	1 (0.4)	2 (0.8)	1 (0.4)	4 (0.5)	
Second-hand smoke exposure	52 (19.7)	49 (18.9)	56 (22.2)	157 (20.3)	0.665
Alcohol use					
Never	195 (73.9)	194 (74.9)	186 (73.8)	575 (74.2)	0.746
Stopped	66 (25.0)	61 (23.6)	61 (24.2)	188 (24.3)	
Still drinking	2 (0.8)	1 (0.4)	4 (1.6)	7 (0.9)	
Calcium supplement intake rate	23 (8.7)	160 (61.8)	204 (81.0)	387 (50.0)	<0.001
Pre-pregnancy BMI (kg/m^2^)	22.0 ± 3.3	21.4 ± 3.4	21.7 ± 3.2	21.7 ± 3.3	0.045
BMI at interview (kg/m^2^)	22.6 ± 3.5	23.3 ± 3.6	26.2 ± 3.3	24.0 ± 3.8	<0.001
Physical activity level					
Low	128 (48.5)	119 (46.0)	77 (30.6)	324 (41.8)	<0.001
Medium	133 (50.4)	137 (52.9)	172 (68.3)	442 (57.0)	
High	3 (1.1)	3 (1.2)	3 (1.2)	9 (1.2)	
DDS	6.09 ± 1.53	6.85 ± 1.51	6.92 ± 1.40	6.61 ± 1.53	<0.001

^a^ Data expressed as the mean ± standard deviation for continuous variables and as *n* (%) for categorical variables. BMI, body mass index; DDS, dietary diversity score.

**Table 2:** Proportions of food group consumption according to the dietary diversity score (DDS).

Food Groups	DDS < 7 (*n* = 354)		DDS ≥ 7 (*n* = 421)		All (*n* = 775)	
	*n*	%	*n*	%	*n*	%
Starchy staples	353	99.7	420	99.8	773	99.7
Flesh foods	291	82.2	413	98.1 ***	704	90.8
Other vegetables	297	83.9	396	94.1 ***	693	89.4
Other fruits	252	71.2	385	91.5 ***	637	82.2
Eggs	166	46.9	339	80.5 ***	505	65.2
Dark green leafy vegetables	142	40.1	305	72.5 ***	447	57.7
Other vitamin A-rich fruits and vegetables	114	32.2	289	68.7 ***	403	52.0
Dairy	100	28.3	293	69.6 ***	393	50.7
Pulses	89	25.1	240	57.0 ***	329	42.5
Nuts and seeds	52	14.7	189	44.9 ***	241	31.1

*** *p* < 0.001.

**Table 3:** Mean nutrient adequacy ratio (NAR) for nutrients according to the dietary diversity score (DDS).

Nutrient	DDS < 7 (*n* = 354)	DDS ≥ 7 (*n* = 421)	All (*n* = 775)	*r* ^a^
Vitamin A	0.53 ± 0.34	0.78 ± 0.25 ***	0.67 ± 0.32	0.443 ***
Thiamin	0.60 ± 0.25	0.75 ± 0.23 ***	0.68 ± 0.25	0.303 ***
Riboflavin	0.58 ± 0.26	0.79 ± 0.21 ***	0.69 ± 0.25	0.457 ***
Niacin	0.84 ± 0.22	0.93 ± 0.14 ***	0.89 ± 0.19	0.289 ***
Vitamin C	0.59 ± 0.34	0.73 ± 0.30 ***	0.66 ± 0.32	0.248 ***
Vitamin E	0.94 ± 0.14	0.97 ± 0.10 ***	0.96 ± 0.12	0.161 ***
Folate	0.40 ± 0.23	0.58 ± 0.24 ***	0.50 ± 0.25	0.384 ***
Calcium	0.47 ± 0.28	0.70 ± 0.25 ***	0.59 ± 0.29	0.447 ***
Phosphorus	0.90 ± 0.18	0.98 ± 0.06 ***	0.94 ± 0.14	0.383 ***
Potassium	0.71 ± 0.25	0.90 ± 0.15 ***	0.81 ± 0.22	0.484 ***
Magnesium	0.70 ± 0.24	0.85 ± 0.17 ***	0.78 ± 0.22	0.402 ***
Iron	0.71 ± 0.24	0.80 ± 0.20 ***	0.76 ± 0.23	0.232 ***
Zinc	0.80 ± 0.22	0.93 ± 0.13 ***	0.87 ± 0.19	0.399 ***
Copper	0.96 ± 0.11	1.00 ± 0.03 ***	0.98 ± 0.08	0.281 ***
Selenium	0.61 ± 0.28	0.74 ± 0.24 ***	0.68 ± 0.27	0.288 ***
MAR ^b^	0.69 ± 0.17	0.83 ± 0.12 ***	0.77 ± 0.16	0.493 ***

^a^ Pearson’s correlation coefficient; ^b^ MAR, mean adequacy ratio; *** *p* < 0.001.

**Table 4:** Bone status according to the dietary diversity score (DDS) in the different trimesters.

	Bone Health	DDS < 7	DDS ≥ 7	All
All population	*n*	354	421	775
	SOS ^a^	4208.2 ± 130.1	4222.4 ± 139.1	4215.9 ± 135.2
	T-score	0.18 ± 1.12	0.31 ± 1.20	0.25 ± 1.17
	Z-score	0.66 ± 1.11	0.75 ± 1.18	0.71 ± 1.15
First trimester	*n*	160	104	264
	SOS	4219.8 ± 140.4	4226.4 ± 142.7	4222.4 ± 141.0
	T-score	0.28 ± 1.21	0.33 ± 1.19	0.30 ± 1.20
	Z-score	0.74 ± 1.17	0.75 ± 1.18	0.75 ± 1.17
Second trimester	*n*	104	155	259
	SOS	4187.9 ± 116.3	4231.4 ± 132.0 **	4213.9 ± 127.5
	T-score	0.00 ± 1.02	0.39 ± 1.16 **	0.24 ± 1.12
	Z-score	0.52 ± 1.02	0.85 ± 1.12 *	0.72 ± 1.09
Third trimester	*n*	90	162	252
	SOS	4211.1 ± 124.8	4211.2 ± 143.5	4211.2 ± 136.8
	T-score	0.22 ± 1.08	0.22 ± 1.25	0.22 ± 1.19
	Z-score	0.67 ± 1.10	0.64 ± 1.24	0.65 ± 1.19

^a^ SOS, speed of sound; * *p* < 0.05; ** *p* < 0.01.

**Table 5:** Linear regression analysis of the association between the dietary diversity score (DDS) and the speed of sound (SOS).

	β (SE)	95% CI	*p*	*R^2^*
All population				
Model 1 ^a^	8.39 (3.17)	(2.18, 14.61)	0.008	0.0090
Model 2 ^b^	8.04 (3.49)	(1.19, 14.90)	0.022	0.0548
Model 3 ^c^	7.68 (3.44)	(0.92, 14.44)	0.026	0.0548
First trimester				
Model 1 ^a^	7.54 (5.67)	(−3.61, 18.70)	0.184	0.0067
Model 2 ^d^	7.85 (6.46)	(−4.88, 20.58)	0.226	0.0859
Model 3 ^c^	4.38 (6.30)	(−8.04, 16.80)	0.488	0.0711
Second trimester				
Model 1 ^a^	16.55 (5.18)	(6.35, 26.75)	0.002	0.0382
Model 2 ^d^	18.07 (5.77)	(6.70, 29.43)	0.002	0.1020
Model 3 ^c^	17.18 (5.69)	(5.97, 28.39)	0.003	0.0990
Third trimester				
Model 1 ^a^	4.44 (6.15)	(−7.68, 16.56)	0.471	0.0021
Model 2 ^d^	2.77 (6.86)	(−10.74, 16.28)	0.687	0.0694
Model 3 ^c^	3.27 (6.70)	(−9.94, 16.48)	0.626	0.0724

^a^ Univariate linear regression of the DDS on the SOS. ^b^ Multivariable adjusted for age, nationality, area, education level, income level, parity, current body mass index, physical activity level, energy intake, smoking status, second-hand smoke exposure, alcohol use and *trimester*. ^c^ Further adjustment for calcium supplement intake. ^d^ Multivariable adjusted for age, nationality, area, education level, income level, parity, current body mass index, physical activity level, energy intake, smoking status, second-hand smoke exposure and alcohol use.

## Data Availability

The data presented in this study are available on request from the corresponding author. The data are not publicly available due to ethical requirements.

## Supplementary Materials

The following supporting information can be downloaded at: https://www.mdpi.com/article/10.3390/nu14214690/s1, Figure S1: Flow chart of sample selection; Table S1: Proportion of food group consumption in the different trimesters; Table S2: Pearson’s correlation coefficients between the NAR and the DDS in the different trimesters.
