# Supplementary material for: Dietary Diversity, Micronutrient Adequacy and Bone Status during Pregnancy: A Study in Urban China from 2019 to 2020
Source: Nutrients. 2022 Nov 5;14(21):4690. doi: 10.3390/nu14214690 (PMC9656709; doi:10.3390/nu14214690)
Supplement: Supplementary file 1 [file nutrients-14-04690-s001.zip › nutrients-2009967-supplementary.pdf]

Dietary Diversity, Micronutrient Adequacy and Bone Status during Pregnancy: A Study in Urban China from 2019 to 2020

Wuxian Zhong [1], Ai Zhao [2], Hanglian Lan [3,4], Shuai Mao [1], Pin Li [1], Hua Jiang [5], Peiyu Wang [6,7,*], Ignatius Man-Yau Szeto [3,4] and Yumei Zhang [1,6,*]

[1] Department of Nutrition and Food Hygiene, School of Public Health, Peking University Health Science Center, Beijing 100191, China
[2] Vanke School of Public Health, Tsinghua University, Beijing 100084, China
[3] Yili Maternal and Infant Nutrition Institute, Beijing 100071, China
[4] Inner Mongolia Dairy Technology Research Institute Co., Ltd., Hohhot 010110, China
[5] School of Nursing, Peking University Health Science Center, Beijing 100191, China
[6] Beijing Key Laboratory of Toxicological Research and Risk Assessment for Food Safety, School of Public Health, Peking University Health Science Center, Beijing 100191, China
[7] Department of Social Medicine and Health Education, School of Public Health, Peking University Health Science Center, Beijing 100191, China
* Correspondence: wpeiyu@bjmu.edu.cn (P.W.); zhangyumei@bjmu.edu.cn (Y.Z.)

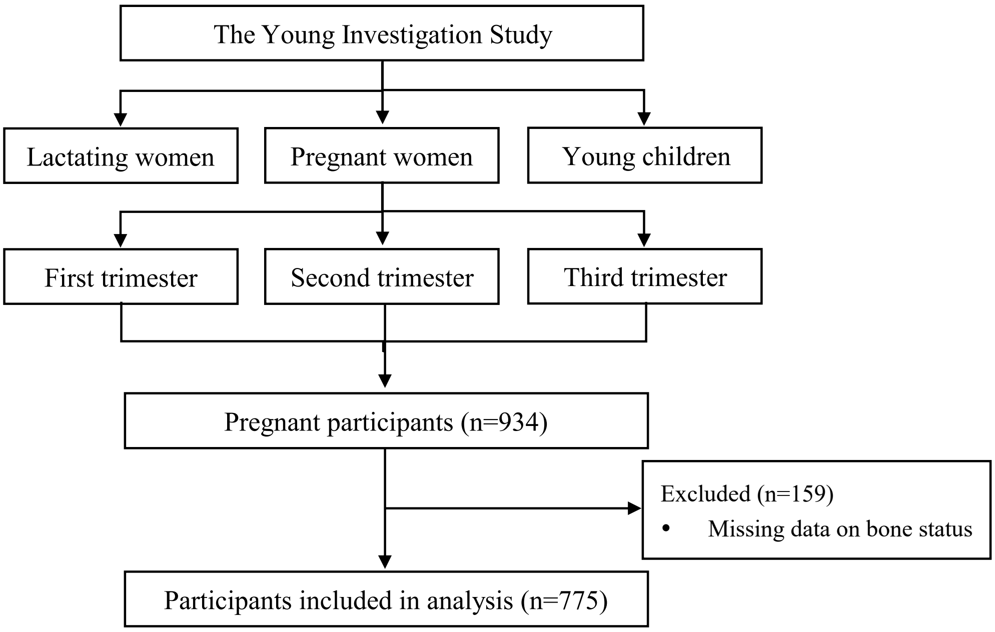

Figure S1. Flow chart of sample selection.

Table S1. Proportion of food group consumption in the different trimesters.

Food group	Trimester 1 (n = 264)		Trimester 2 (n = 259)		Trimester 3 (n = 252)		p
	n	%	n	%	n	%	
Starchy staples	264	100.0	258	99.6	251	99.6	0.550
Pulses	104	39.4	112	43.2	113	44.8	0.435
Nuts and seeds	76	28.8	92	35.5	73	29.0	0.169
Dairy	91	34.5	144	55.6	158	62.7	<0.001
Flesh foods	221	83.7	246	95.0	237	94.1	<0.001
Eggs	152	57.6	174	67.2	179	71.0	0.004
Dark green leafy vegetables	140	53.0	146	56.4	161	63.9	0.039
Other vitamin A-rich fruits and vegetables	126	47.7	141	54.4	136	54.0	0.230
Other vegetables	232	87.9	234	90.4	227	90.1	0.602
Other fruits	202	76.5	227	87.6	208	82.5	0.004

Table S2. Pearson's correlation coefficients between the NAR and the DDS in the different trimesters [a].

NAR for each nutrient	First trimester	Second trimester	Third trimester
Vitamin A	0.512	0.335	0.391
Thiamin	0.280	0.272	0.354
Riboflavin	0.486	0.395	0.426
Niacin	0.294	0.146	0.319
Vitamin C	0.219	0.208	0.273
Vitamin E	0.108	0.180	0.175
Folate	0.384	0.317	0.389
Calcium	0.462	0.406	0.416
Phosphorus	0.370	0.327	0.391
Potassium	0.478	0.381	0.486
Magnesium	0.346	0.350	0.423
Iron	0.287	0.174	0.317
Zinc	0.376	0.284	0.423
Copper	0.303	0.218	0.299
Selenium	0.314	0.145	0.289
MAR	0.488	0.410	0.502

[a] All p values < 0.001. MAR, mean adequacy ratio.
